# Betacellulin regulates the proliferation and differentiation of retinal progenitor cells *in vitro*

**DOI:** 10.1111/jcmm.13321

**Published:** 2017-09-18

**Authors:** Dandan Zhang, Bingqiao Shen, Yi Zhang, Ni Ni, Yuyao Wang, Xianqun Fan, Hao Sun, Ping Gu

**Affiliations:** ^1^ Department of Ophthalmology Ninth People's Hospital Shanghai Jiao Tong University School of Medicine Shanghai China

**Keywords:** betacellulin, retinal progenitor cell, proliferation, differentiation

## Abstract

Retinal progenitor cells (RPCs) hold great potential for the treatment of retinal degenerative diseases. However, their proliferation capacity and differentiation potential towards specific retinal neurons are limited, which limit their future clinical applications. Thus, it is important to improve the RPCs’ ability to proliferate and differentiate. Currently, epidermal growth factor (EGF) is commonly used to stimulate RPC growth *in vitro*. In this study, we find that betacellulin (BTC), a member of the EGF family, plays important roles in the proliferation and differentiation of RPCs. Our results showed that BTC can significantly promote the proliferation of RPCs more efficiently than EGF. EGF stimulated RPC proliferation through the EGFR/ErbB2‐Erk pathway, while BTC stimulated RPC proliferation more powerfully through the EGFR/ErbB2/ErbB4‐Akt/Erk pathway. Meanwhile, under differentiated conditions, the BTC‐pre‐treated RPCs were preferentially differentiated into retinal neurons, including photoreceptors, one of the most important types of cells for retinal cell replacement therapy, compared to the EGF‐pre‐treated RPCs. In addition, knockdown of endogenous BTC expression can also obviously promote RPC differentiation into retinal neuronal cells. This data demonstrate that BTC plays important roles in promoting RPC proliferation and differentiation into retinal neurons. This study may provide new insights into the study of RPC proliferation and differentiation and make a step towards the application of RPCs in the treatment of retinal degenerative diseases.

## Introduction

Retinal degenerative diseases, including retinitis pigmentosa and age‐related macular degeneration, are threats to human health 1. RPCs, a group of cells isolated from the retina that retain self‐renewal capabilities and differentiate into both neuronal and glial cells, show promise for use in cell replacement therapy for treating retinal degenerative diseases 2, 3. However, the efficient expansion and differentiation of RPCs into retinal neurons are the subject of ongoing studies 4, 5, 6. On the one hand, RPCs are relatively difficult to obtain, and cultured RPCs have a very limited proliferation capacity, which typically cannot meet clinical needs 7, 8. To efficiently obtain a sufficient number of cells for RPC research and to investigate their potential applications in cell replacement therapy for retinal degenerative diseases, it is worth exploring the mechanisms involved in RPC proliferation to ultimately find ways to improve the proliferation capacity of RPCs 9. On the other hand, in addition to self‐renewal, these progenitors can give rise to all of the major cell types in the neural retina, including retinal neurons and glia, *via* multilineage differentiation 10. However, the obstacles with regard to RPC differentiation include RPCs’ limited differentiation capacity and preferential differentiation into the glial lineage rather than the neuronal lineage, which ultimately restricts the application of RPCs 11, 12. However, if the proliferation and efficient differentiation problems of RPCs were improved or clarified, RPCs would become one of the best seed cells for the cell replacement treatment of retinal degenerative diseases.

Epidermal growth factor (EGF) is well known to regulate the proliferation and self‐renewal of neural stem cells (NSCs) 13, 14. EGF and the EGF‐like ligands/EGF receptors activate multiple intracellular pathways and are believed to promote the proliferation and self‐renewal of NSCs 15, 16. Currently, four EGF receptors have been identified: ErbB1 (EGFR), ErbB2 (HER2/neu), ErbB3 (HER3) and ErbB4 (HER4) 17. After being activated by EGF or EGF‐like ligands, the inactivated monomers dimerize in the form of homodimers or heterodimers, followed by the autophosphorylation of their intracellular domain. The phosphorylated receptor then initiates a cascade of intracellular events 18. Retinal progenitors are a type of neural stem cells, and it is known that RPCs proliferate and remain undifferentiated in serum‐free culture medium in the presence of EGF 2, 4, 5, 12, 19. However, RPCs have a very limited proliferation capacity in serum‐free culture medium containing EGF. Furthermore, the efficiency of the EGF‐pre‐treated RPCs’ differentiation into retinal neuronal cells is also limited. It has been reported that the presence of EGF in the culture medium may specifically influence the RPC differentiation potential to promote gliogenesis 20, 21. Thus, it is worth exploring alternative culture conditions that can promote RPC proliferation in a better way and can effectively regulate RPC differentiation into neurons.

Betacellulin (BTC), a member of the EGF family, is a 32 KD glycoprotein that was first discovered in the conditioned medium of murine beta‐cell carcinoma 22. BTC promotes the proliferation of a wide variety of cell types, including pancreatic cells and vascular smooth muscle cells 23, 24, 25. As an EGF‐like ligand, BTC works in similar but somewhat different ways than EGF, as it was reported to generally bind to the ErbB1 and ErbB4 homodimers and to the ErbB1/ErbB2, ErbB1/ErbB3, ErbB1/ErbB4, ErbB2/ErbB3 and ErbB2/ErbB4 heterodimers in many other cell types 26. The Akt and Erk pathways are the two primary pathways that are induced by BTC/ErbBs. However, the detailed mechanisms and downstream targets may not be the same in different cells 23, 24. Specifically, in neural stem cells, BTC activates both the Erk and Akt pathways, whereas EGF mainly activates the Erk pathway, which ultimately promotes neural stem cell proliferation 27. However, little is known about the effects of BTC on RPC proliferation or the related mechanisms.

BTC also acts as a differentiation factor for pancreatic tumour cells 28. Furthermore, in terms of stem cells, BTC is known to induce mesenchymal stem cell (MSC) differentiation into the pancreatic lineage 29. BTC can mediate embryonic pancreas differentiation 30, and it is known to induce the differentiation of undifferentiated embryonic stem cells (ESCs) into an intermediate, multi‐potential, neuronal‐like population 31 and to stimulate neural stem cells to differentiate into neuronal cells 27. However, it remains unknown whether BTC contributes to the regulation of RPC differentiation.

The effects of BTC on RPC proliferation and differentiation were investigated in this study. Our results showed that BTC was an efficient cytokine that induced RPC proliferation. Studies of the related mechanisms showed that BTC activates ErbB tyrosine kinase receptors *via* the ErbB1, ErbB2 and ErbB4 subunits of EGFR, and that it evokes more Erk phosphorylation than EGF phosphorylation while also evoking Akt phosphorylation, ultimately resulting in a much stronger stimulation of RPC proliferation compared to EGF. Meanwhile, our data demonstrate that the BTC‐pre‐treated RPCs preferentially differentiate into retinal neuronal cells compared to the EGF‐pre‐treated RPCs. These results indicate that BTC plays a key role in governing RPC proliferation and differentiation.

## Materials and methods

### Isolation and culture of murine retinal progenitor cells (RPCs) and culture system description

The RPCs were isolated from fresh neural retina of post‐natal day 1 C57BL/6 mice that were genetically modified with GFP (a kind gift from Dr. Masaru Okabe, University of Osaka, Japan) 32. Subsequently, the RPCs were plated in T25 flasks in fresh proliferation medium consisting of advanced DMEM/F12 (Invitrogen, Carlsbad, CA, USA), 1% N2 neural supplement (Invitrogen), 2 mM L‐glutamine (Invitrogen) and 20 ng/ml of recombinant (EGF, Invitrogen). To detect the proliferation ability of the RPCs, they were plated in T25 flasks in fresh proliferation medium consisting of advanced DMEM/F12 (Invitrogen), 1% N2 neural supplement (Invitrogen), 2 mM L‐glutamine (Invitrogen) and 20 ng/ml of recombinant EGF (Invitrogen) or different concentration gradients of recombinant (BTC, Peprotech, Rocky Hill, NJ, USA). The cells were cultured for 3 days (unless otherwise indicated) and then collected for qPCR, Western blot analysis or immunocytochemistry analysis. To detect the instantaneous expression level or phosphorylation level of receptors or downstream targets induced by BTC, cells were cultured in the aforementioned medium for 20 min. To detect the long‐term effect of BTC on RPC receptor expression, cells were cultured in the medium for 10 days (with normal passage intervals). For RPC differentiation analysis, the cells were cultured with differentiation media consisting of 10% foetal bovine serum (FBS) (Invitrogen) without EGF. To detect the effects of BTC on RPC differentiation, RPCs were cultured in medium consisting of advanced DMEM/F12 (Invitrogen), 1% N2 neural supplement (Invitrogen), 2 mM L‐glutamine (Invitrogen), 10% FBS (Invitrogen) and 20 ng/ml of BTC (Peprotech). To detect the differentiation potential influence of BTC on RPCs, the cells were first cultured in medium consisting of advanced DMEM/F12 1% N2 neural supplement, 2 mM L‐glutamine and 20 ng/ml of EGF or 20 ng/ml BTC for 10 days with regular passage intervals. Then, BTC or EGF was withdrawn, 10% FBS was added, and the cells were further cultured for 7 day for differentiation. To knockdown the endogenous expression of BTC, the RPCs were expanded in traditional culture medium with the addition of EGF. Then, the cells were allowed to differentiate and adhere in traditional differentiation medium with EGF withdrawn for 1 day. The siBTC oligonucleotides were transfected every 3 days thereafter. All animals used in this study were prudently handled according to the ARVO animal usage standards and received approval from the Animal Care and Use Committee of the Schepens Eye Research Institute, where the cells were originally derived.

### Quantification of the cells’ morphology and viability

To assess the RPC morphology during seeding in BTC or EGF cultures (proliferation state), the same concentration of RPCs was seeded at 0 hr, and the cell morphology was observed under a fluorescence microscope (Olympus BX51, Olympus, Japan) at 24, 48 and 72 hrs after seeding.

The cell density of the RPCs was the same for all conditions at the time of seeding. First, RPCs were seeded in 96‐well plates and incubated in a medium without any cytokines (BTC or EGF). The number of viable cells was determined using a CCK‐8 assay kit (Dojindo, Kumamoto, Japan). Then, to get the standard curve of the RPCs in 20 ng/ml EGF, 5000, 10,000, 20,000, 40,000 and 80,000 cells per well were seeded separately in the standard medium (including 20 ng/ml EGF), and the number of viable cells was determined using a CCK‐8 assay kit (Dojindo). Finally, the RPCs (10,000 cell per well) were seeded in 96‐well plates and incubated with proliferation medium containing BTC (2.5, 5,10, 20 and 40 ng/ml), EGF (20 ng/ml), BTC plus AG1478 (Sigma‐Aldrich, Saint Louis, MO, USA; 3 nM), BTC plus AG825 (Sigma‐Aldrich; 0.35 μM), BTC plus LY294002 (Sigma‐Aldrich, 1.4 μM), BTC plus PD98059 (Sigma‐Aldrich, 10 μM) and BTC plus DMSO (control) (Sigma‐Aldrich). The number of viable cells was determined using a CCK‐8 assay kit (Dojindo). The absorbance of each well was measured at 450 nm using a microplate reader (ELX800, BioTek, Winooski, Vermont, USA) at 0, 24, 48 and 72 hrs of cell culture.

### Live–dead viability assay

To determine the effect of the inhibitors (AG1478, AG825, LY294002 and PD98059) on RPC viability, a calcein acetoxymethyl ester (CAM)/ethidium homodimer 1 (EH‐1) (Invitrogen) assay was performed. In brief, RPCs were cultured for 3 days in standard culture medium in the presence of AG1478, AG825, LY294002 and PD98059 or not (the control group). Then, cells were incubated in PBS containing 2 mM CAM and 2 mM EH‐1 for 20 min. at 37°C, and the cell morphologies were observed under a fluorescence microscope (Olympus BX51).

### Mouse phospho‐RTK array

The RPCs were washed with phosphate‐buffered saline (PBS), starved for 1 hr in DMEM/F12 medium and incubated with 20 ng/ml BTC or EGF for 20 min. Then, the total cellular proteins were harvested, and a BCA kit (Pierce, Rockford, IL, USA) was used to analyse the protein concentrations. Approximately 150 μg of the protein samples was loaded onto each nitrocellulose membrane. A mouse Phospho‐RTK Array Kit, which contains 39 fixed antibodies against the phosphorylated forms of tyrosine kinases, was used according to the manufacturer's instructions (R&D Systems, Inc., Minneapolis, MN, USA). The grey‐scale images were analysed with ImageJ (http://rsbweb.nih.gov/ij/). Each kinase array dot was manually selected, and an average intensity for each kinase was calculated. Normalization within one stimulation experiment was performed by averaging the grey values of the two repeats and then subtracting the intensity of the negative control dot (PBS) from each value.

### Detection of the differentiation and differentiation potential of BTC on retinal progenitor cells *in vitro*

To clarify whether the BTC additive in the differentiation medium (medium with 10% FBS) has an effect on RPC differentiation, Western blot analyses were performed. To detect the effects of BTC or EGF pre‐treatment on RPC differentiation, the RPCs underwent 10 days of individual incubation with BTC or EGF. Then, they were collected without digestion and were cultured in differentiation medium (Invitrogen) for the indicated times. The medium was refreshed at intervals of 2 or 3 days. Images of the cells’ morphology were captured with a fluorescence microscope (Olympus IX51) on days 10, 14 and 17. The cells were then collected for total RNA or protein extraction on day 17.

### Transfection

The small interfering RNAs were synthesized by Biomics Biotech Co., Ltd. (Nantong, China). RPCs were seeded in differentiation medium for the differentiation and transfection experiments. These were conducted on the second day because the RPCs had attached to the plate by that point. To transfect the RNA duplexes, a 20 nM final concentration of siRNA was mixed with Lipofectamine 2000 (Invitrogen) in Opti‐MEM serum‐free medium and incubated for 20 min. at room temperature 4. The mixture was then added to the cell cultures and was replaced by differentiation medium 8 hrs later. For long‐term detection under differentiation culture conditions, the siBTC oligonucleotides were repeatedly transfected every 3 days. The oligonucleotide sequence of siBTC was 5′‐CAUUACUGCAUCCAUGGGAdTdT‐3′.

### RNA isolation, reverse transcription and quantitative polymerase chain reaction (qPCR)

Total RNA was extracted from each sample of cultured cells with Trizol reagent (Invitrogen). The extracted total RNA concentration was measured by spectrophotometer and a nanodrop 2000 software, and the qualified purity levels were assessed by the OD260/280 nm ratios between 1.9 and 2.1. Then, 1000 nanogram of qualified RNA was reverse‐transcribed with the PrimeScript™ RT reagent kit (Perfect Real Time; TaKaRa, Dalian, China). qPCR was performed in a 10‐μl total volume containing 5 μl of 2× Power SYBR Green PCR Master Mix (Applied Biosystems, Irvine, CA, USA), 1 μl of diluted cDNA and 150 nM of gene‐specific primers, and the remaining volume was composed of nuclease‐free water (Invitrogen). The primer sequences are shown in Table 1. The PCR efficiency of the reaction was measured with primers using serial dilutions of the cDNA (1:1, 1:5, 1:25, 1:125, 1:625 and 1:3125). Then, the samples were amplified with a 7500 Real‐Time PCR Detection System (Applied Biosystems). After 40 cycles of amplification, the relative mRNA was analysed using the Pfaffl method 33. The relative mRNA levels were expressed as the fold change relative to the negative controls after normalization to the expression of housekeeping gene β‐actin.

**Table 1:** Primers used for quantitative RT‐PCR

Genes	Forward (5′–3′)	Forward(5–3′)	Annealing temperature (°C)	Product size (base pairs)
P27	ccagacgtaaacagctccgaatta	aggcagatggtttaagagtgcc	60	194
Nestin	aactggcacctcaagatgt	tcaagggtattaggcaagggg	60	235
Vimentin	tggttgacacccactcaaaa	gcttttggggtgtcagttgt	60	132
Pax6	ttaaactctggggcaggtcc	gtgtcaggtgagtctggtgg	60	112
β3‐tubulin	cgagacctactgcatcgaca	cattgagctgaccagggaat	60	152
Map2	agaaaatggaagaaggaatgactg	acatggatcatctggtaccttttt	60	112
Recoverin	atggggaatagcaagagcgg	gagtccgggaaaaacttggaata	60	179
Rhodopsin	tcaccaccaccctctacaca	tgatccaggtgaagaccaca	60	216
PKCα	cccattccagaaggagatga	ttcctgtcagcaagcatcac	60	212
GFAP	agaaaaccgcatcaccattc	tcacatcaccacgtccttgt	60	184
AP2α	gccgtccacctagccaggga	gattgggccgcgagttcccc	60	208
Calbindin	tcaggatggcaacggataca	aataagagcaaggtctgttcgg	60	169
CRALBP	agggtctttgttcacggagat	tgccactagagcgttcctaaa	60	297
β‐actin	agccatgtacgtagccatcc	ctctcagctgtggtggtgaa	60	152

### Immunocytochemistry

The RPCs were seeded onto 13‐mm laminin‐coated (Sigma‐Aldrich) glass coverslips (VWR, West Chester, PA, USA) in 24‐well plates after digestion. After 3 days of culture in the proliferation medium or 7 days of culture in the differentiation medium, the cells were rinsed with warm PBS, fixed with 4% paraformaldehyde (Sigma‐Aldrich) for 15 min., permeabilized with 0.3% Triton X‐100 (Sigma‐Aldrich) and blocked with 10% normal goat serum (Invitrogen) for 1 hr. Next, the cells were incubated with mouse monoclonal anti‐Ki‐67 (1:200; BD, San Jose, CA, USA), mouse monoclonal anti‐Nestin (1:200; Millipore, Billerica, MA, USA), rabbit monoclonal anti‐Vimentin (1:200; Millipore), rabbit polyclonal anti‐Pax6 (1:200; Millipore), mouse monoclonal anti‐β3‐tubulin (1:100; Millipore), rabbit polyclonal anti‐Recoverin (Millipore, 1:200), mouse monoclonal anti‐cellular retinaldehyde‐binding protein (CRALBP) (1:200; Abcam, Cambridge, MA, USA), mouse monoclonal anti‐activator protein 2 α (AP2α, 1:200; Developmental Studies Hybridoma Bank(DSHB); Iowa City, IA, USA), mouse monoclonal anti‐cone‐rod‐box (CRX, 1:200; Novus, CO, USA) and mouse monoclonal antiglial fibrillary acidic protein (GFAP, 1:200; Millipore) antibodies overnight at 4°C. The cells were incubated with a 1:800 dilution of fluorescently labelled secondary antibodies in PBS (Alexa Fluor546‐goat antimouse/rabbit, BD) in the dark. After three rinses in PBS, the cell nuclei were then counterstained with 4′,6‐diamidino‐2‐phenylindole (DAPI; Invitrogen). Negative controls were performed simultaneously using the same protocol, with the exception of the incubation with the primary antibody. The immunoreactive cells were then visualized and imaged with a fluorescence microscope (Olympus BX51). Meanwhile, the percentage of positive cells was calculated using the Image‐Pro Plus 6.0 software (Media Cybernetics, Bethesda, MD, USA). For 5‐bromo‐2‐deoxyuridine (BrdU) incorporation, 10 mM BrdU (Sigma‐Aldrich) was used. After 12 hrs, the proliferating cells were detected using a monoclonal anti‐BrdU antibody (Cell Signaling Technology (CST), Danvers, MA, USA).

### Western blot analysis

The total cellular proteins were harvested at the specified time‐points. A BCA kit (Pierce) was used to analyse the protein concentrations. Then, a 25 μg protein sample was loaded onto the sodium dodecyl sulphate‐polyacrylamide gels (SDS‐PAGE) and electrophoresed to separate the proteins. Subsequently, the proteins were transferred to 0.22 lm polyvinylidene fluoride membranes (Millipore). Next, the membranes were blocked with 5% BSA and then incubated with rabbit antiphospho‐Tyr (CST, 1:1000), rabbit monoclonal anti‐EGFR (Santa Cruz Biotechnology, Santa Cruz, CA, USA, 1:200), rabbit monoclonal anti‐ErbB2 (Abcam; 1:500), rabbit monoclonal anti‐ErbB4 (Santa Cruz Biotechnology, 1:200), rabbit monoclonal antiphospho‐EGFR (CST, 1:1000), rabbit monoclonal antiphospho‐ErbB2 (CST, 1:1000), rabbit monoclonal antiphospho‐ErbB4 (CST, 1:1000), rabbit monoclonal anti‐Akt (CST, 1:1000), rabbit monoclonal anti‐P‐Akt (CST, 1:1000), rabbit monoclonal anti‐Erk (CST,1:1000), rabbit monoclonal anti‐P‐Erk (CST, 1:1000), mouse monoclonal anti‐Nestin (BD, 1:500), rabbit monoclonal anti‐Vimentin (Millipore, 1:1000), rabbit polyclonal anti‐Pax6 (Millipore, 1:1000), mouse monoclonal anti‐Rhodopsin (Millipore, 1:200), mouse monoclonal antiprotein kinase C‐alpha (PKC‐α) (Millipore, 1:200), mouse monoclonal anti‐β3‐tubulin (Millipore, 1:1000), mouse monoclonal anti‐GFAP (Millipore, 1:1000), rabbit polyclonal anti‐Recoverin (Millipore, 1:1000), mouse monoclonal anti‐CRALBP (1:200; Abcam), goat polyclonal antibetacellulin (Santa Cruz Biotechnology, 1:200) and mouse anti‐β‐actin (Sigma‐Aldrich, 1:5000) antibodies overnight at 4°C. Then, after incubation with DyLightTM680‐conjugated secondary antibodies (Sigma‐Aldrich, 1:5000), the protein expression levels were observed using an Odyssey V 3.0 image scanner (LI‐COR, Lincoln, NE, USA). The intensities of the protein bands were densitometrically quantified using the BandScan 5.0 software, and the values for each sample were standardized against those of β‐actin.

### Statistical analyses

The experimental statistics presented in this study are expressed as the mean ± standard derivation (S.D.) All experiments were performed in triplicate unless otherwise specified. Statistical analyses were performed with Student's *t*‐test, and the difference was considered significant at *P* ≤ 0.05.

## Results

### BTC promotes RPC proliferation

**Figure 1:**
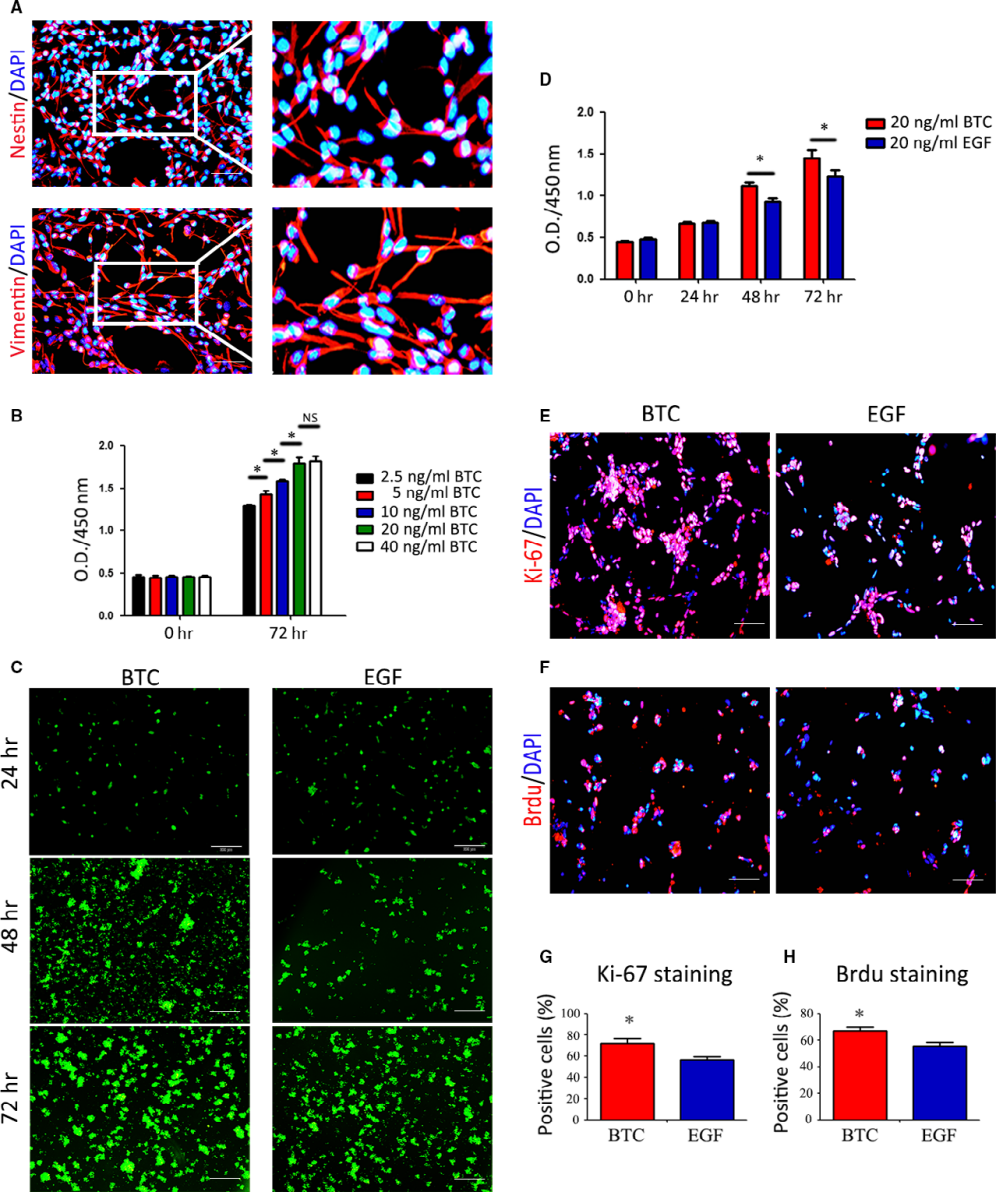
BTC promotes RPC proliferation**.** (**A**) Isolation and identification of RPCs by immunocytochemistry. More than 80% of the cultured cells were Nestin‐ and Vimentin‐positive. (**B**) Detection of the effect of different concentrations of BTC on RPC proliferation by CCK‐8 analysis. (**C**) Fluorescent micrographs of the GFP+ RPCs cultured in 20 ng/ml BTC or 20 ng/ml EGF for the indicated times. The cell clusters (the green fluorescence) in the 20 ng/ml BTC cultures were larger than the RPC clusters in 20 ng/ml EGF cultures at 48 and 72 hrs. (**D**) CCK‐8 analysis of RPC proliferation in the presence of 20 ng/ml BTC or 20 ng/ml EGF. The O.D. 450 values at 48 and 72 hrs were significantly increased in BTC‐treated group compared to the EGF‐treated group. (**E**–**H**) The immunocytochemistry analysis of the percentages of Ki‐67‐positive cells and BrdU‐positive cells in the RPC cultures treated with 20 ng/ml BTC or 20 ng/ml EGF showed that the percentages were markedly increased in the RPC cultures treated with 20 ng/ml BTC compared to the 20 ng/ml EGF group. *P < 0.05 by Student's *t*‐test. Scale bars: A: 100 μm; C: 200 μm. E‐F: 100 μm.

BTC promotes the proliferation of a variety of cell types 24, 26, but the effect of BTC on RPC proliferation was unclear. RPCs were isolated from the fresh retinal tissue of post‐natal day 1 GFP‐transgenic C57BL/6 mice as previously reported 19. As shown in Figure 1A, more than 80% of the cells in the RPC cultures were positive for Nestin (a general marker for retinal progenitors) and Vimentin (a marker for retinal progenitors) expression, which was consistent with previous reports (Fig. 1A). As shown in Figure S1, RPCs that were cultured in medium without any cytokines cannot sustain their proliferation (Fig. S1). The O.D. values of different concentrations of RPCs cultured in standard medium (containing 20 ng/ml EGF) were tested, and the standard curves are shown in Figure S2. The results showed that the O.D values had a linear relationship with the concentration of RPCs varying from 5000 to 80,000 per well. To investigate the effects of BTC on RPC proliferation, the cells were cultured in medium containing BTC and not EGF. The CCK‐8 analysis, which was used to detect proliferation, demonstrated that BTC is a powerful and efficient cytokine with the ability to induce RPC proliferation (Fig. 1B). BTC stimulated the proliferation of RPC in a concentration‐dependent manner. The expansion capacity of the RPCs was gradually enhanced when the concentrations of BTC varied from 2.5 to 20 ng/ml. However, when the concentration increased to 40 ng/ml, the proliferation capacity of the RPCs did not obviously increase compared to the cells treated with 20 ng/ml. Thus, a concentration of 20 ng/ml BTC was used in this study. As shown in Figure 1C, the RPCs were cultured with 20 ng/ml BTC or EGF for the indicated times, and the cell clusters (green fluorescence) in the 20 ng/ml BTC cultures were larger than the RPC clusters in the 20 ng/ml EGF cultures at both 48 and 72 hrs (Fig. 1C). Corresponding phase pictures are shown in Figure S3. Moreover, the results of the CCK‐8 analysis showed a similar trend. At 48 and 72 hrs, the O.D. 450 value was higher in the BTC‐treated group than in the EGF‐treated group (Fig. 1D). In addition, Ki‐67 immunostaining and 5‐bromodeoxyuridine (BrdU) analyses were also used to evaluate the proliferation capacity of the RPCs. The results showed that the ratios of Ki‐67‐ (71.95 ± 7.85% and 56.28 ± 5.64%, for BTC and EGF, respectively) and BrdU‐positive (67 ± 5.06% and 55.49 ± 4.83%, for BTC and EGF, respectively) cells were increased in the BTC‐treated cultures compared to the EGF‐treated cultures (Fig. 1E–H). Moreover, the expression levels of p27, a marker of cell cycle kinase inhibitor, were evaluated, which indicated that when exposed to BTC for 3 days or as long as 10 days (passage 3 times), P27 was down‐regulated compared to the EGF‐treated group (to the extent of 0.69 ± 0.12‐fold at day 3 and 0.61 ± 0.24‐fold at day 10) (Fig. S4). These results indicate that BTC promotes RPC proliferation better than EGF does, which is important for obtaining a large number of cells.

### BTC promotes the proliferation of RPCs by targeting the membrane receptors EGFR, ErbB2 and ErbB4

**Figure 2:**
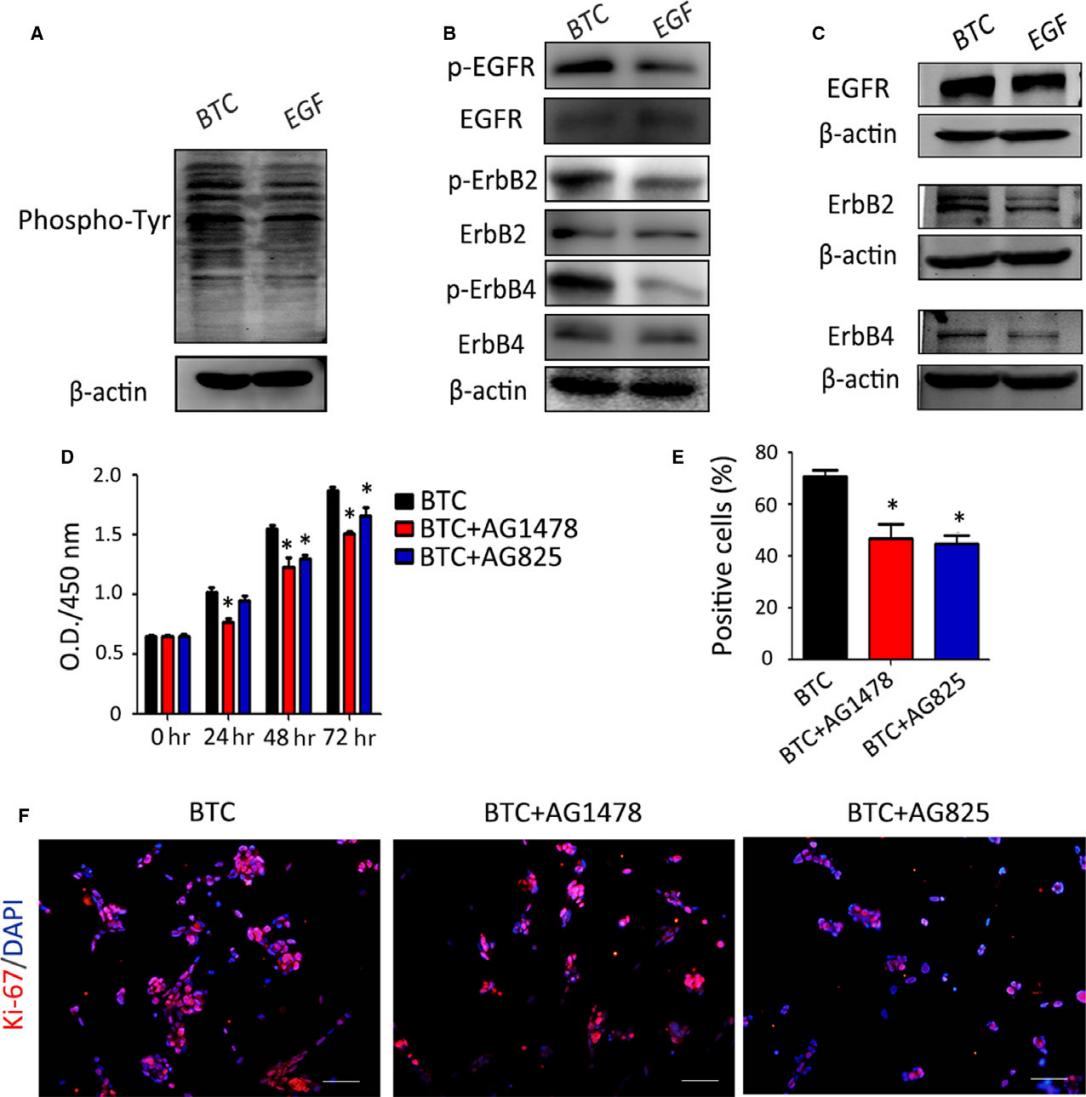
BTC promotes the proliferation of RPCs through targeting the membrane receptors EGFR, ErbB2 and ErbB4. (**A**) Detection of the levels of tyrosine phosphorylation in the RPCs that were treated with 20 ng/ml BTC or 20 ng/ml EGF for 20 min. by Western blot analysis. The results showed that BTC caused more tyrosine phosphorylation. (**B**) A Western blot analysis of effect of 20 min. treatment of BTC and EGF on the expression levels of phospho‐EGFR, phospho‐ErbB2 and phospho‐ErbB4 was performed, and the phosphorylation levels of EGFR, ErbB2 and ErbB4 in the BTC‐treated group were increased compared to the EGF‐treated group. (**C**) A Western blot analysis of effect of long‐time treatment of BTC and EGF on the expression levels of EGFR, ErbB2 and ErbB4 was performed, and the expression levels of EGFR, ErbB2 and ErbB4 in the BTC‐treated group were increased compared to the EGF‐treated group. (**D**–**F**) The RPCs were stimulated with BTC (20 ng/ml) in the presence of chemical inhibitors of EGFR and ErbB4 (AG1478), chemical inhibitors of ErbB2 (AG825) or DMSO (BTC groups), and the RPCs’ growth was measured by CCK‐8 analysis (**D**) and Ki‐67 immunostaining (**E**–**F**). *P < 0.05 by Student's *t*‐test. Scale bars: 100 μm.

In NSCs, it was reported that BTC, an EGF‐like ligand, functions *via* EGF family receptors, followed by the autophosphorylation of the tyrosine residues of their intracellular domains to initiate a cascade of intracellular events. To investigate the mechanisms by which BTC and EGF evoke RPC proliferation, the RPCs were cultured in the presence of 20 ng/ml BTC or 20 ng/ml EGF, and the level of tyrosine phosphorylation was determined by Western blot analysis, which showed that tyrosine phosphorylation can be detected in the presence of BTC or EGF. Furthermore, BTC induced more tyrosine phosphorylation than EGF (Fig. 2A). Because BTC and EGF exert their biological effects through the ErbB receptors, a mouse phosphoreceptor tyrosine kinases (RTK) protein array was performed to determine the distribution of the ErbB receptor subtypes in RPCs and the corresponding subtypes that were involved in BTC‐ or EGF‐induced proliferation. As shown in Fig. S5, both BTC and EGF activated the phosphorylation of EGFR, ErbB2 and ErbB4 at 20 min., but BTC stimulated a stronger phosphorylation of EGFR, ErbB2 and ErbB4 compared to EGF (approximately 1.28‐fold, 1.54‐fold and 3.13‐fold, respectively). Western blot analysis was used to further confirm that the BTC stimulated a stronger phosphorylation of EGFR, ErbB2 and ErbB4 at 20 min. (Fig. 2B). Moreover, to determine whether the long‐term treatment of RPCs in BTC can affect the total amount of the corresponding transmembrane receptors, RPCs were treated with BTC or EGF for 10 days, and the expression levels of EGFR, ErbB2 and ErbB4 were evaluated by Western blot analysis. Increased expression levels of EGFR, ErbB2 and ErbB4 were detected in the BTC‐treated group (Fig. 2C). The above‐mentioned clues suggest that BTC evoked prompt and strong tyrosine phosphorylation of the EGF family receptors EGFR, ErbB2 and ErbB4, leading to an up‐regulation of the total expression levels of EGFR, ErbB2 and ErbB4 in long‐term treatment. However, it was not clear whether the BTC‐induced expression and phosphorylation of EGFR, ErbB2 and ErbB4 correlated with the BTC‐induced RPC proliferation. Thus, AG1478 (a chemical inhibitor of EGFR and ErbB4) and AG825 (a chemical inhibitor of ErbB2) were added to the BTC‐treated cultures, and the results of the CCK‐8 analysis showed that the BTC‐induced RPC proliferation can be partially abolished by AG1478 or AG825 (Fig. 2D). Moreover, the immunocytochemistry analysis displayed consistent results, showing that the ratio of Ki‐67‐positive cells was 46.54 ± 9.75% in the AG1478 group and 44.62 ± 5.66% in AG825 group, which was lower than the BTC cultures (70.67 ± 4.46%) (Fig. 2E–F). In addition, to prove that the decreased O.D. values obtained in the CCK8 analysis in the presence of inhibitors had nothing to do with the direct cytotoxity of inhibitors on RPCs, a LIVE/DEAD staining of the RPCs that proliferated after 3 days in standard medium with the presence of inhibitors was performed, and the results showed no difference between the inhibitor groups and the control group (Fig. S6). These results indicate that BTC stimulates the expansion of RPCs *via* the transmembrane receptors EGFR, ErbB2 and ErbB4 and that when these receptors are inhibited, the growth‐promoting effects of BTC are subsequently attenuated.

### BTC promotes RPC proliferation by targeting the Akt and Erk signalling pathways downstream of the EGF receptor family

**Figure 3:**
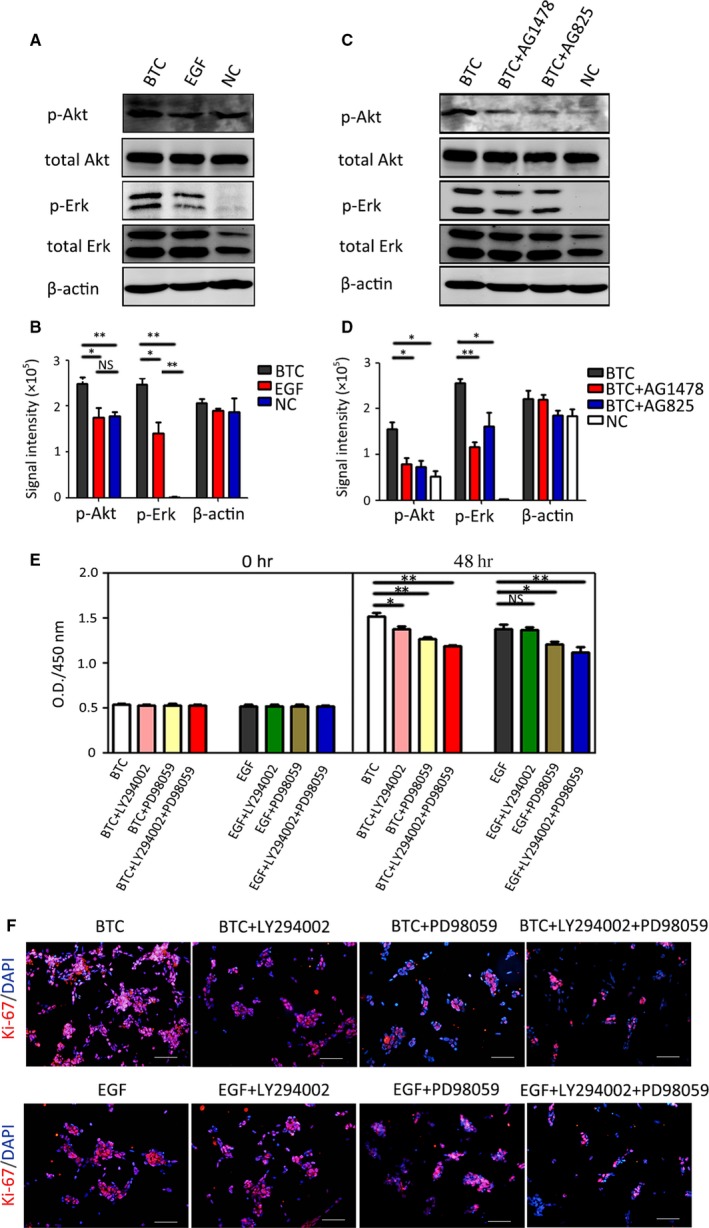
BTC promotes RPC proliferation by targeting the AKT and Erk signalling pathways downstream of the EGF receptor family. (**A**–**B**) The RPCs were not stimulated (NC) or were stimulated with EGF (20 ng/ml) or BTC (20 ng/ml) for 20 min., and the protein extracts were assessed by Western blot analysis using antibodies against specific phosphorylated residues (p‐Akt and p‐Erk) or the total protein (Akt and Erk). The results showed that BTC caused stronger phosphorylation of Akt and Erk. (**C**–**D**) The RPCs were not stimulated with BTC (NC) or were stimulated with BTC (20 ng/ml) in the presence of AG1478 (chemical inhibitors of EGFR and ErbB4) or AG825 (chemical inhibitors of ErbB2) for 20 min., and the protein extracts were assessed by Western blot analysis using antibodies against specific phosphorylated residues (p‐Akt and p‐Erk) or the total protein (Akt and Erk). (**E**–**F**) RPCs were stimulated with BTC (20 ng/ml) or EGF (20 ng/ml) in the presence of LY294002 (chemical inhibitor of Akt), PD98059 (chemical inhibitor of Erk) or LY294002 plus PD98059, and the RPCs’ growth was measured by CCK‐8 analysis (**E**) and Ki‐67 immunostaining (**F**). *P < 0.05, **P < 0.01 by Student's *t*‐test. Scale bars: 100 μm.

BTC and EGF were demonstrated to activate different ErbB receptors in this study, and the downstream pathways that were involved in BTC‐ and EGF‐induced RPC proliferation were further investigated. Generally, growth factor receptors may mediate the activation of intracellular PI3‐kinase/Akt and MEK/Erk cascades, which induce cell proliferation. Therefore, the effects of BTC and EGF on Akt (Ser473) and Erk1/2 phosphorylation were examined in cultured RPCs, and the Western blot results showed that compared to the control group (RPCs without any cytokines), EGF induced Erk phosphorylation but barely induced Akt phosphorylation. BTC induced Erk and Akt phosphorylation more than the control group did. In addition, BTC stimulated more Akt and Erk phosphorylation compared to EGF (Fig. 3A–B). Moreover, the BTC‐induced Akt and Erk phosphorylation levels were attenuated when the transmembrane receptors EGFR and ErbB4 were inhibited with AG1478 or when ErbB2 was inhibited with AG825. These results indicate that Akt and Erk are the downstream targets of EGFR, ErbB2 and ErbB4 (Fig. 3C–D). Thus, BTC may promote the proliferation of RPCs through the EGFR/ErbB2/ErbB4‐Akt/Erk pathway. To determine whether Akt and Erk phosphorylation contribute to BTC‐ or EGF‐induced proliferation, CCK‐8 and immunocytochemistry analyses were performed. The results showed that both LY294002 (the chemical inhibitor of Akt) and PD98059 (the chemical inhibitor of Erk) can reduce BTC‐induced RPC proliferation (Fig. 3E–F). Of these, PD98059 can impair RPC proliferation more powerfully than LY294002 in the EGF‐treated cultures, which suggests that EGF‐induced proliferation mainly relies on Erk phosphorylation. However, in the BTC‐treated cultures, both PD98059 and LY294002 can obviously impair RPC proliferation, which indicates that both Akt and Erk have important roles in BTC‐evoked RPC proliferation. Taken together, our data demonstrate that BTC is capable of inducing the expansion of RPCs. We found that this effect is mediated by EGFR, ErbB2 and ErbB4, which, in turn, activate the Erk and Akt signalling pathways. At the same time, BTC is more powerful in activating Erk than EGF is, and BTC can evoke Akt phosphorylation, but EGF did not. This may partially explain why BTC induces RPC proliferation more effectively than EGF.

### BTC can keep RPCs in a progenitor state under proliferation conditions like EGF did

**Figure 4:**
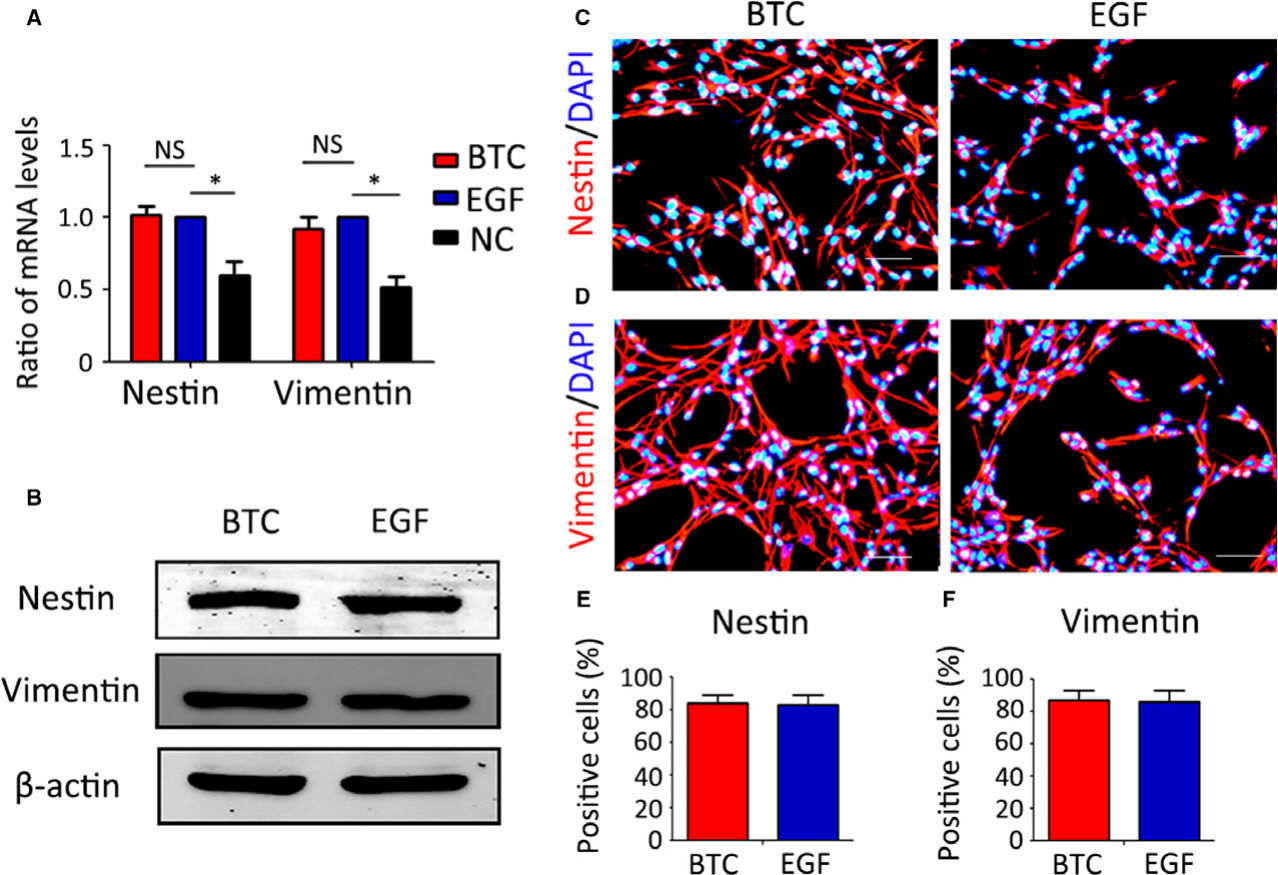
BTC can keep RPCs in a progenitor state under proliferation conditions like EGF did. (**A**) The expression levels of Nestin and Vimentin (markers of RPCs) in the RPCs cultured with 20 ng/ml BTC or 20 ng/ml EGF or no cytokines (NC) were evaluated by qPCR analysis, and the results showed that there were no obvious difference between the two groups. (**B**) Western blot analysis of the expression levels of Nestin and Vimentin in the RPCs cultured with 20 ng/ml BTC or 20 ng/ml EGF. (**C**–**F**) Immunocytochemistry analysis of the ratios of Nestin‐ and Vimentin‐positive cells in the RPCs cultured with 20 ng/ml BTC or 20 ng/ml EGF. The results showed that there were no remarkable differences between the two groups. Scale bars: 50 μm.

We investigated whether BTC can keep RPCs undifferentiated in proliferation conditions as EGF did. qPCR results showed that the expression levels of Nestin, Vimentin and Pax6 in the RPCs were comparable in the BTC‐treated cultures and EGF‐treated cultures, while the expression level of Nestin and Vimentin in both groups was higher than that in the no cytokines group (Fig. 4A, Fig. S7A). Western blot results (Fig. 4B, Fig. S7B) showed that the expression levels of Nestin, Vimentin and Pax6 in the RPCs were comparable in the BTC‐treated cultures and EGF‐treated cultures. Moreover, the immunocytochemistry analysis displayed similar results, showing that the ratios of Nestin‐positive (84.0 ± 6.67% and 83.0 ± 8.0%, for the BTC and EGF groups, respectively) and Vimentin‐positive (87.0 ± 7.33% and 85.7 ± 9.1% and Pax6‐positive cells (65.9 ± 8.4% and 61.3 ± 11.4%), for the BTC and EGF groups, respectively) in the BTC‐treated cultures were comparable to those in the EGF‐treated cultures (Fig. 4C–F, Fig. S7C). To evaluate the differentiation state of the BTC‐ or EGF‐treated cells, we assessed the β3‐tubulin, Rhodopsin, CRX and CRALBP expression levels through immunocytochemical analyses. The results showed the negative expression of these markers in the proliferation state (Fig. S8), which suggests that BTC, similar to EGF, can maintain the RPCs in progenitor state under proliferation conditions.

### The differentiation of RPCs was weakened by the direct addition of BTC into the differentiation medium, whereas the pre‐treatment of RPC with BTC can enhance the differentiation potential of RPCs

To determine whether BTC has an effect on RPC differentiation when added directly to the differentiation medium (medium with 10% FBS), Western blot analysis was performed. The results showed that the direct addition of BTC to the differentiation medium weakened the differentiation ability of the RPCs (Fig. S9).

**Figure 5:**
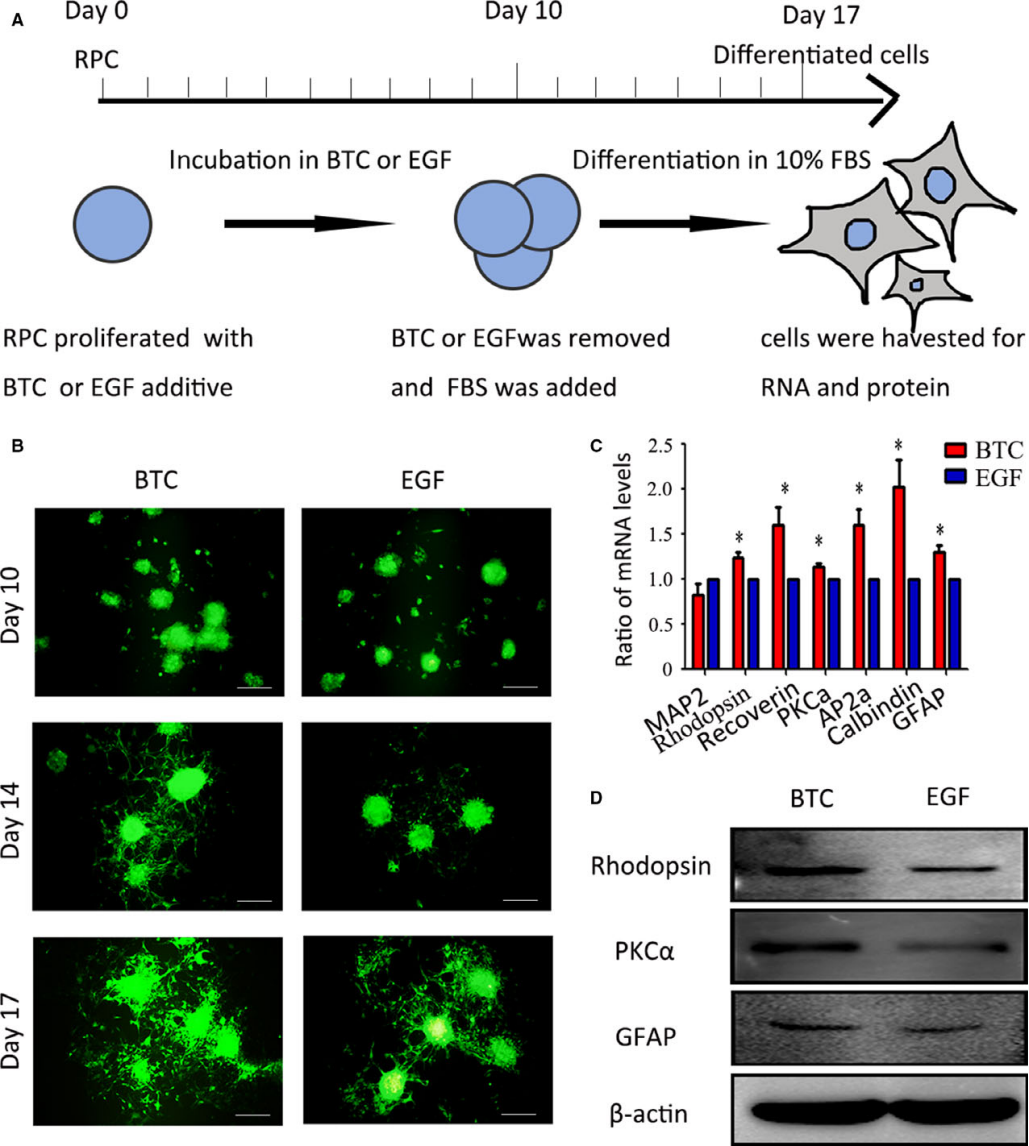
The RPCs’ differentiation potential towards retinal neurons was enhanced by the BTC pre‐treatment. (**A**) Schematic diagram of the detection of the RPCs’ differentiation potential. (**B**) The cells were investigated on days 10, 14 and 17 to compare the differentiation potential of the BTC‐ and EGF‐pre‐treated RPC cultures. The results showed that the BTC‐pre‐treated RPC cultures (day 14 and day 17) exhibited longer neurites. (**C**) The qPCR results showed that the expression level of MAP2 in the BTC‐pre‐treated RPC cultures was comparable to that of the EGF‐pre‐treated group, while the expression levels of Rhodopsin, Recoverin, PKC‐α, AP2‐α, Calbindin and GFAP were obviously increased compared to the EGF‐pre‐treated group. (**D**) Western blot analysis showed enhanced expression of Rhodopsin, PKC‐α and GFAP. *P < 0.05 by Student's *t*‐test. Scale bars: 200 μm.

However, we found that BTC pre‐treatment can enhance RPCs’ differentiation potential into retinal neurons. As shown in Figure 5A, the RPCs were cultured in proliferation medium with BTC or EGF for 10 days. Then, BTC or EGF was removed, and the culture medium was replaced with a differentiation medium containing 10% FBS for an additional 7 days for differentiation. The cells were investigated on days 10, 14 and 17 to compare the differentiation potential of the BTC‐ and EGF‐pre‐treated RPC cultures. Green fluorescence was indicative of RPCs, and the results showed that BTC‐pre‐treated RPC cultures (days 14 and 17) exhibited longer neurites (Fig. 5B). Corresponding phase pictures are shown in Fig. S3B. It is well known that EGF‐pre‐treated RPCs can differentiate into different retinal neuronal and glial marker‐positive cells. Such markers include MAP2 (a marker for neurons), Rhodopsin (a marker for rod photoreceptors), Recoverin (a marker for rod and corn photoreceptors), PKC‐α (a marker for bipolar cells), AP2α (a marker for amacrine cells), Calbindin (a maker for horizontal cells) and GFAP (a glial marker). In this study, qPCR and Western blot analyses were performed to investigate the potential fates of the BTC‐pre‐treated RPCs, and the results showed that the BTC‐pre‐treated RPCs were able to differentiate into retinal neuron lineages (Fig. 5C–D). The qPCR results showed that the expression levels of MAP2 in the BTC‐treated cultures were comparable to those in the EGF‐treated group, while the expression levels of Rhodopsin, Recoverin, PKC‐α, AP2‐α, Calbindin and GFAP were obviously increased compared to the EGF‐treated cells (by approximately 1.23‐fold, 1.6‐fold, 1.14‐fold, 1.6‐fold, 2.02‐fold and 1.29‐fold, respectively) (Fig 5C). The Western blot analysis showed that the expression of Rhodopsin, PKC‐α and GFAP was enhanced (Fig 5D). The immunocytochemistry analysis results showed that the ratios of β3‐tubulin (a marker for neurons) (37.60 ± 4.76% and 23.13 ± 4.29%, respectively), AP2α (11.70 ± 2.88% and 5.3 ± 2.56%, respectively) and Recoverin (17.2 ± 2.55% and 8.97 ± 2.10%, respectively) were significantly increased in the BTC‐pre‐treated cells compared to the control cultures (Fig. S10). These data suggest that the BTC‐pre‐treated RPCs can differentiate towards their retinal neuron lineages. This represents an interesting for future clinical applications, including cell replacement therapy for retinal degenerative diseases.

### The knockdown of endogenous BTC promotes RPC differentiation

**Figure 6:**
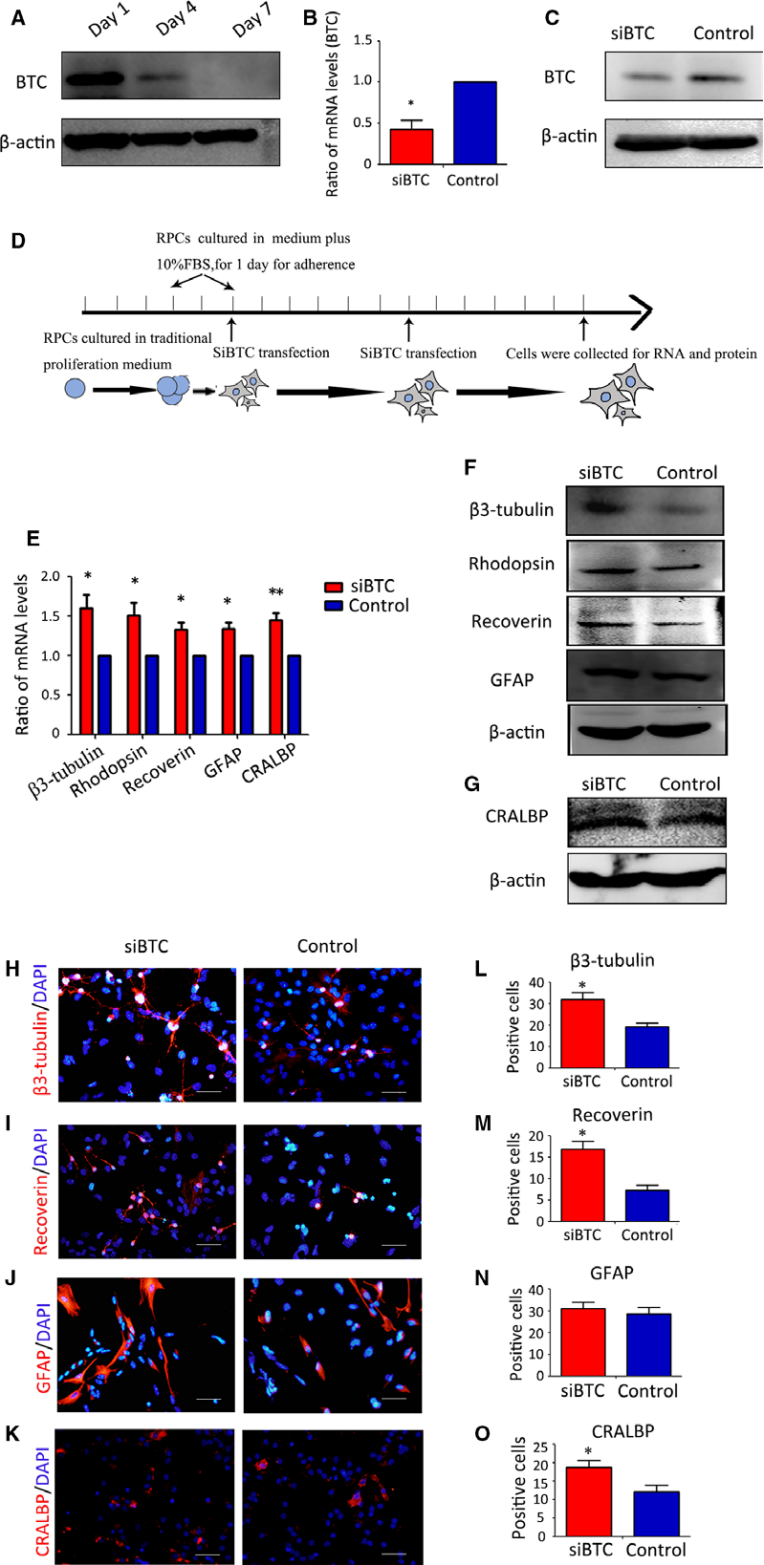
Knockdown of endogenous BTC could promote RPC differentiation**.** (**A**) Western blot analysis showed that the endogenous expression of BTC was gradually down‐regulated in the RPC cultures during the differentiation process. (**B**–**C**) The qPCR and Western blot results revealed that siBTC obviously down‐regulated the expression level of BTC. (**D**) schematic diagram of the knockdown experiments. (**E**) qPCR analysis of the effects of siBTC on RPC differentiation. The results showed that the siBTC‐transfected RPC cultures up‐regulated β3‐tubulin, Rhodopsin, Recoverin, GFAP and CRALBP expression compared to the control group. (**F**–**G**) Western blot analysis showed that the expression levels of β3‐tubulin, Rhodopsin, Recoverin, GFAP and CRALBP were obviously increased in the siBTC‐treated RPC cultures compared to control group. (**H**–**K**) After 7 days of culture under differentiation conditions, the siBTC‐transfected or untransfected (control) RPCs were immunostained for β3‐tubulinn, rhodopsin, GFAP and CRALBP, as indicated. (**L**–**O**) The percentages of β3‐tubulin‐, rhodopsin‐ and CRALBP‐positive cells were significantly increased, whereas the percentage of GFAP‐positive cells was not significantly different in the siBTC group compared to the control group. Notes: The bars represent the means ± standard deviation (*n* = 3); *P < 0.05 by Student's *t*‐test. Scale bars: 50 μm.

Because BTC is known to be expressed in many cell types, we were interested in studying the endogenous expression of BTC during the RPC differentiation process. A Western blot analysis showed that BTC expression was gradually down‐regulated during the RPC differentiation process (Fig. 6A), indicating that endogenous BTC may play an important role in regulating RPC differentiation. To explore the effects of endogenous BTC on RPC differentiation, siBTC was transfected into the RPCs, and the knockdown efficiency of BTC was measured by qPCR and Western blot analysis. The results showed that the expression of BTC was repressed by more than 60% (Fig. 6B–C). A schematic diagram of the knockdown experiments is shown in Figure 6D. The results of the qPCR analysis showed that the siBTC‐transfected RPC cultures up‐regulated β3‐tubulin, Rhodopsin, Recoverin, GFAP and CRALBP (a marker for müller cells) expression (by approximately 1.59‐fold, 1.51‐fold, 1.33‐fold, 1.34‐fold and 1.45‐fold, respectively) compared to the control group (Fig. 6E). Furthermore, the Western blot analysis showed that the expression levels of β3‐tubulin, Rhodopsin, Recoverin, GFAP and CRALBP were markedly increased in the siBTC‐treated RPC cultures compared to the control group (Fig. 6F and G). In addition, the immunocytochemistry results showed the ratios of β3‐tubulin (31.93 ± 5.5% and 19.1 ± 3.05%, respectively), Recoverin (16.86 ± 3.11% and 7.23 ± 2.13%, respectively) and CRALBP (37.60 ± 4.76% and 23.13 ± 4.29%, respectively) were significantly increased in the siBTC‐transfected cells compared to the control cultures, which is consistent with the qPCR and Western blot results. In contrast, the ratio of GFAP‐positive cells did not increase significantly (28.76 ± 2.81% and 28.6 ± 4.95%, respectively) (Fig. 6H–O). These data demonstrate that the knockdown of endogenous BTC in the RPCs could promote RPC differentiation. Furthermore, protein expression levels were detected by combining pre‐treatment with the knockdown of BTC on day 10 (differentiation), showing that the expression levels of Rhodopsin, PKC‐α and β3‐tubulin in the combined group were obviously up‐regulated compared to the siBTC group and the NC group (Fig. S11). These data showed that BTC regulation may have important roles in RPC differentiation.

In summary, we demonstrated that BTC can promote RPC proliferation much better than EGF and that BTC‐pre‐treated RPCs preferentially differentiate into specific retinal neurons of interest *in vitro*. Moreover, the knockdown of endogenous BTC expression can obviously promote RPC differentiation. These results suggest that BTC has potential applications in RPC proliferation and differentiation.

## Discussion

RPCs are a group of cells that are characterized by self‐renewal and differentiation into a range of different retinal neuronal and glial cells 3, 34. RPCs have potentially broad applications for the treatment of retinal degenerative diseases such as retinitis pigmentosa and age‐related macular degeneration 3, 35. However, the development of efficient and reliable methods for the *in vitro* expansion and efficient differentiation of RPCs is a significant barrier to the clinical application of RPCs for the treatment of retinal degenerative diseases 36, 37. On the one hand, it is important to further explore the mechanisms involved in the proliferation of RPCs to efficiently obtain a sufficient number of cells for RPC research and RPC applications in retinal cell replacement therapy in the future. On the other hand, for the final application of RPCs, the obstacles with regard to RPC differentiation, including their limited differentiation capacity and preferential differentiation into the glial lineage instead of the neuronal lineage, are hot areas of RPC research. Thus, it is important to find ways to improve RPC proliferation and differentiation.

It is known that RPCs proliferate and remain undifferentiated in serum‐free culture medium in the presence of EGF 5, 7, 38. However, little is known about the mechanisms involved in the process of EGF‐promoted RPC proliferation. In this study, we demonstrated that EGF, an admitted cytokine that induces RPC proliferation, mainly activates RPC proliferation *via* EGFR and ErbB2 phosphorylation and the downstream Erk pathway but induces little phosphorylation of Akt, which was consistent with a previous study on NSCs 27. Moreover, it was reported that the presence of EGF in the culture medium may influence the RPCs’ differentiation potential, which is known to promote gliogenesis 20, 21. Thus, it is necessary to search for alternative or supplemental factor(s) that may better promote RPC proliferation and effectively regulate RPC differentiation into retinal neurons instead of glia.

BTC, a member of the EGF family, was reported to play important roles in NSC proliferation and differentiation 27. During embryogenesis, the retina is derived from the forebrain and thus belongs to the central nervous system (CNS) 39. These findings suggest that BTC may also have specific roles in RPC proliferation or differentiation. Fortunately, our results suggest that BTC significantly promotes RPC proliferation and plays an important role in RPC fate determination. In the current study, our data demonstrate that BTC promotes RPC proliferation more powerfully than EGF does. The reasons for this may be because BTC induced the clear phosphorylation of EGFR, ErbB2 and ErbB4 in RPCs, whereas EGF induced less phosphorylation of EGFR, ErbB2 and ErbB4 in RPCs. Moreover, compared to EGF, BTC up‐regulated the total protein levels of EGFR, ErbB2 and ErbB4, and these receptors are thought to contribute to RPC proliferation as a result of their overexpression 40. Finally, BTC evoked more Erk phosphorylation than EGF did and could also evoke Akt phosphorylation, which may partially explain its stronger growth‐promoting effect on RPCs 41, 42.

In addition, BTC is involved in controlling RPC fate determination. Compared to EGF, the BTC‐pre‐treated RPCs were more inclined to differentiate into retinal neuronal cells, including photoreceptors. The reason may be as follows: BTC has been demonstrated to be a differentiation factor for many cell types, BTC‐induced differentiation cannot be reproduced by applying a similar dose of EGF 29, 31; In addition, previous studies have demonstrated that EGF‐like ligands may induce the phosphorylation of several cell cycle‐related genes, some of which are important for differentiation 43; It was previously demonstrated that the expression levels of EGF receptors contribute to the regulation of cell fate 44, 45, and in this study, BTC and EGF evoked different EGFR/ErbB2/ErbB4 expression levels. Therefore, this may be one reason for the differences in cell fate.

Thus, BTC is a cytokine for inducing RPC growth, not only because it better promotes RPC proliferation compared to EGF but also because it significantly enhances RPCs’ differentiation into specific retinal neurons of interest *in vitro*.

Moreover, BTC was previously demonstrated to be expressed in the pancreas, small intestine, blood vessels, choroid plexus and other tissues 24, 27, 46. In this study, we discovered that endogenous BTC is also expressed in RPCs. With the gradual differentiation of RPCs, the expression of BTC was down‐regulated. The inhibition of endogenous BTC expression by an siRNA resulted in the enhanced differentiation of RPCs into retinal neurons, including photoreceptors, which are one of the most important retinal neuronal cells in retinal cell replacement therapy 47. Although the mechanisms by which BTC expression regulates RPC differentiation remain to be further explored, the retinal microenvironment, in conjunction with intracellular cues, may have an effect on promoting differentiation 48.

In this study, our results suggest that BTC in the proliferation medium can enhance RPC proliferation; however, when we add BTC directly into the traditional differentiation medium, BTC showed to inhibit RPC differentiation. While after being exposed to proliferation medium containing BTC, and then let it differentiate in traditional differentiation medium (without BTC), RPCs’ differentiation potential towards neuronal cells was enhanced. The above‐mentioned results suggest that BTC is similar to, but better than EGF, which can maintain the proliferation and sustain the progenitors’ characteristics of RPCs, meanwhile BTC could promote the differentiation potential of RPCs. Previous studies demonstrated that the expression levels of EGF receptors contribute to the regulation of cell fate 44, 45. In this study, we speculate that different EGFR/ErbB2/ErbB4 expression levels triggered by BTC or EGF tend to be one of the reasons for the differences in cell fate. Moreover, knockdown endogenous expression of BTC can promote RPC differentiation, which was consistent with the above‐mentioned clues (when we add BTC directly into the traditional differentiation medium, BTC showed to inhibit RPC differentiation). In a word, BTC promotes RPCs’ proliferation and their differentiation potential towards neuronal cells, while inhibits RPCs’ differentiation. We expect that further investigations of the mechanisms may open new opportunities to the differentiation regulation and fate determination of RPCs.

## Conclusions

In this study, our data demonstrate that BTC can markedly promote RPC expansion through the EGFR/ErbB2/ErbB4‐Akt/Erk pathway. And BTC pre‐treated RPCs showed an enhanced differentiation ability compared to EGF, including differentiation into photoreceptors, the most important class of retinal neuronal cells for retinal cell replacement therapy. In addition, knockdown of endogenous BTC expression can obviously promote RPC differentiation. The present study of the effects of BTC on RPCs may provide us with some new insights into the controlled proliferation and differentiation of RPCs *in vitro*, but the effect of BTC on RPCs *in vivo* requires further investigation.

## Conflict of interests

The authors declare no competing financial interests.

## Supporting information


**Figure S1** CCK8 analysis of RPC proliferation in medium without any cytokines.
**Figure S2** Standard curve of CCK8 test on mRPCs.
**Figure S3** Phase picture of RPCs cultured in medium for proliferation and differentiation.
**Figure S4** qPCR analysis of P27 expression of RPCs.
**Figure S5** Antibody array detection of the receptor tyrosine kinase phosphorylation.
**Figure S6** LIVE/DEAD staining of RPC proliferate 3 days in standard medium with the presence of AG1478, AG825, LY294002 and PD98059.
**Figure S7** Detection of the expression of Pax6 in BTC treated RPCs.
**Figure S8** Imunocytochemystry analysis of the differentiation state of the BTC or EGF treated cells in proliferation condition.
**Figure S9** Western blot analysis of RPCs’ differentiation influenced by BTC directly adding in the differentiation medium.
**Figure S10** Immunocytochemistry analysis of BTC pretreatment on RPC differentiation.
**Figure S11** Western blot analysis of BTC pretreatment combined with knockdown of BTC on RPC differentiation.
