# Supplementary material for: Betacellulin regulates the proliferation and differentiation of retinal progenitor cells in vitro
Source: J Cell Mol Med. 2017 Sep 18;22(1):330–45. doi: 10.1111/jcmm.13321 (PMC5742713; doi:10.1111/jcmm.13321)
Supplement: Supplementary file 1 — Figure S1 CCK8 analysis of RPC proliferation in medium without any cytokines. Figure S2 Standard curve of CCK8 test on mRPCs. Figure S3 Phase picture of RPCs cultured in medium for proliferation and differentiation. Figure S4 qPCR analysis of P27 expression of RPCs. Figure S5 Antibody array detection of the receptor tyrosine kinase phosphorylation. Figure S6 LIVE/DEAD staining of RPC proliferate 3 days in standard medium with the presence of AG1478, AG825, LY294002 and PD98059. Figure S7 Detection of the expression of Pax6 in BTC treated RPCs. Figure S8 Imunocytochemystry analysis of the differentiation state of the BTC or EGF treated cells in proliferation condition. Figure S9 Western blot analysis of RPCs’ differentiation influenced by BTC directly adding in the differentiation medium. Figure S10 Immunocytochemistry analysis of BTC pretreatment on RPC differentiation. Figure S11 Western blot analysis of BTC pretreatment combined with knockdown of BTC on RPC differentiation. [file JCMM-22-330-s001.docx]

**Betacellulin regulates the proliferation and differentiation of retinal progenitor cells in vitro.**

Dandan Zhang†, Bingqiao Shen†, Yi Zhang†, Ni Ni, Yuyao Wang, Xianqun Fan*, Hao Sun*, and Ping Gu*

Department of Ophthalmology, Ninth People's Hospital, Shanghai Jiao Tong University School of Medicine, Shanghai, 200011, P.R. China

† These authors contributed equally to this work.

* To whom correspondence should be addressed:

Tel: +86 021 2327 1699*5587 Fax: +86 021 6313 7148

Figure. S1. CCK8 analysis of RPC proliferation in medium without any cytokines.


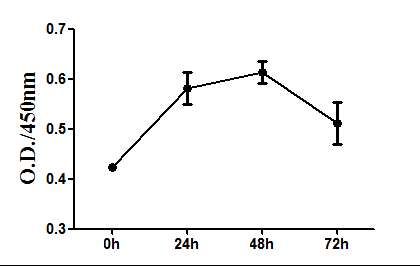


Figure. S2. Standard curve of CCK8 test on mRPCs.


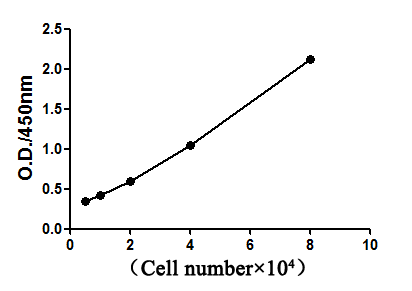


Figure. S3. Phase picture of RPCs cultured in medium for proliferation and differentiation.


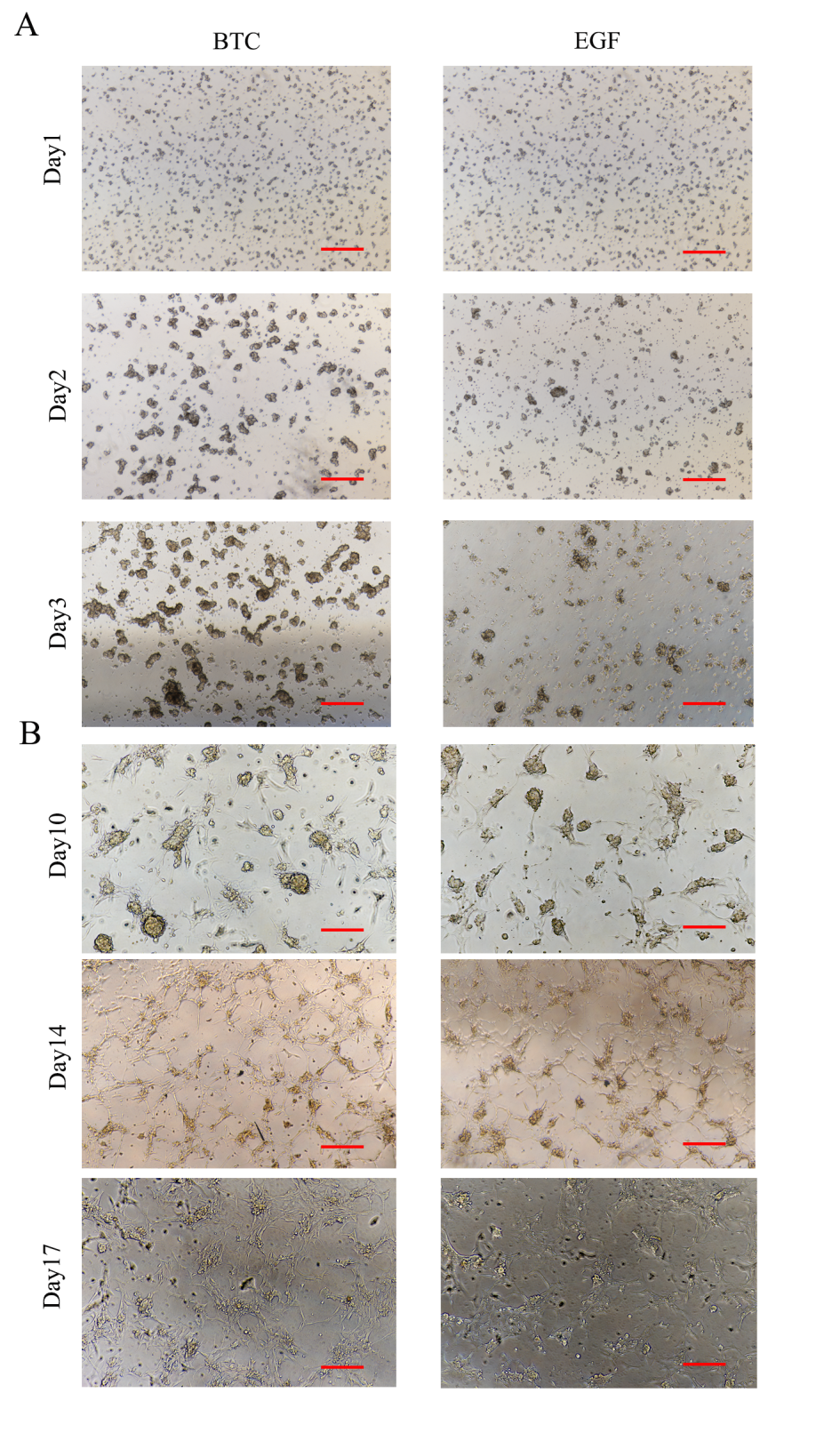


Figure. S4. qPCR analysis of P27 expression of RPCs.


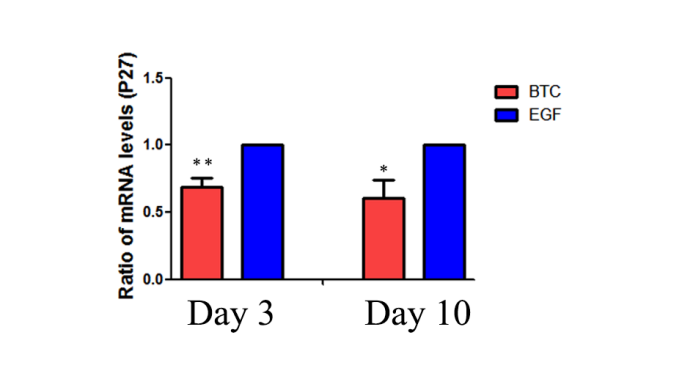


Figure. S5. Antibody array detection of the receptor tyrosine kinase phosphorylation.


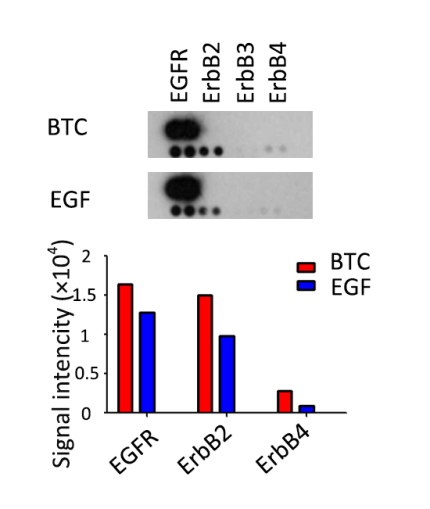


Figure. S6. LIVE/DEAD staining of RPC proliferate 3 days in standard medium with the presence of AG1478, AG825, LY294002 and PD98059.


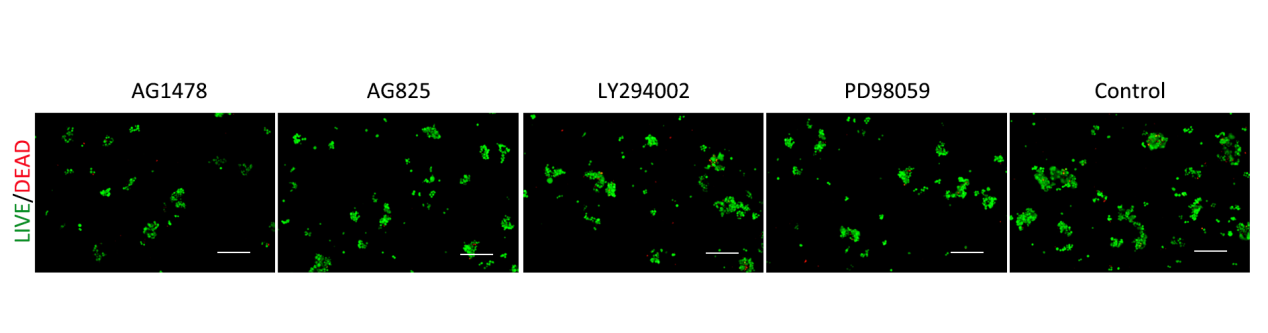


Figure. S7. Detection of the expression of Pax6 in BTC treated RPCs .
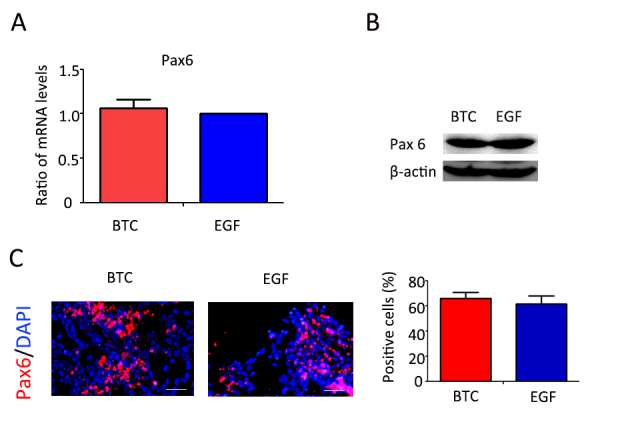


Figure. S8. Imunocytochemystry analysis of the differentiation state of the BTC or EGF treated cells in proliferation condition.
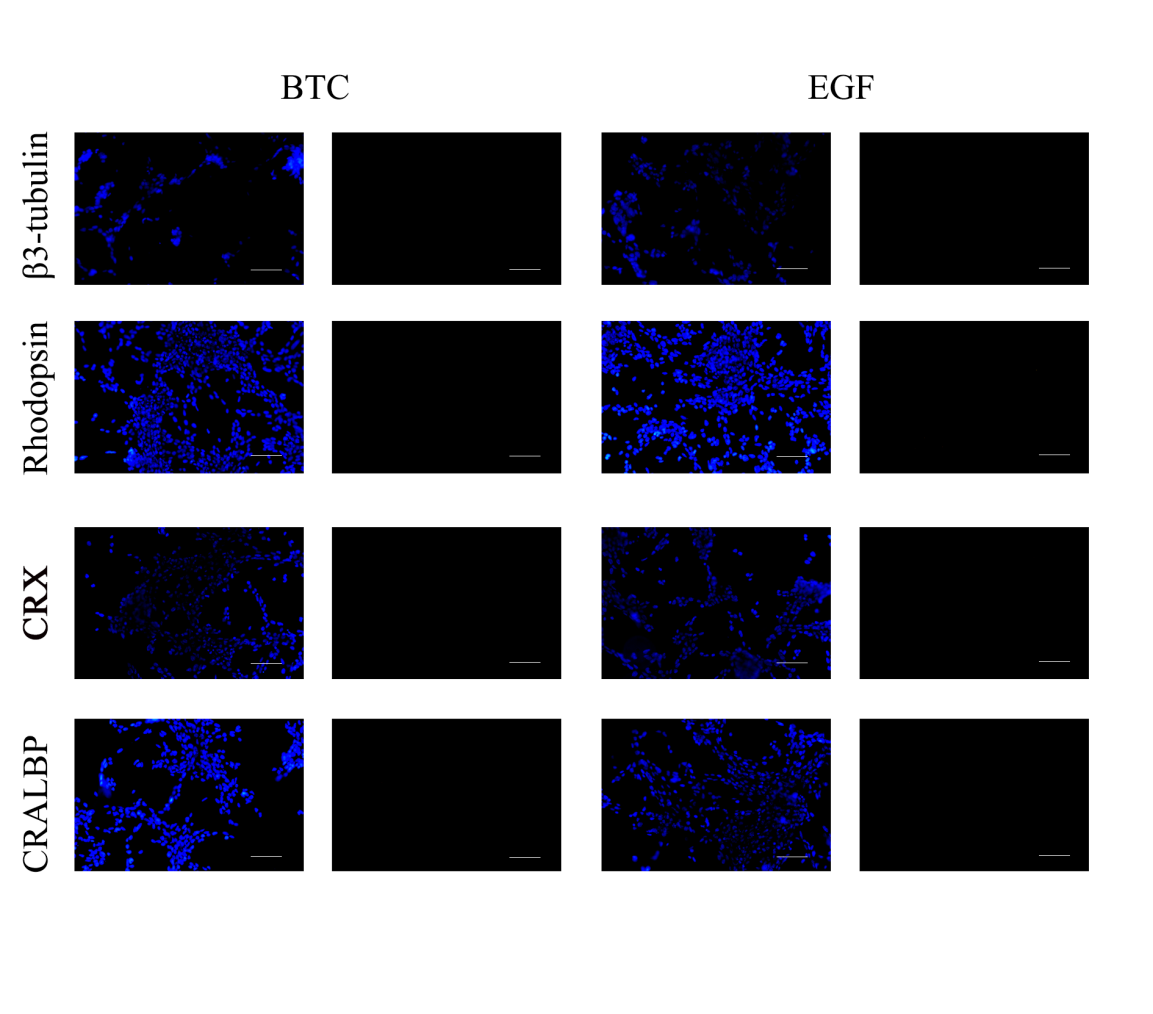


Figure. S9. Western blot analysis of RPCs’ differentiation influenced by BTC directly adding in the differentiation medium.


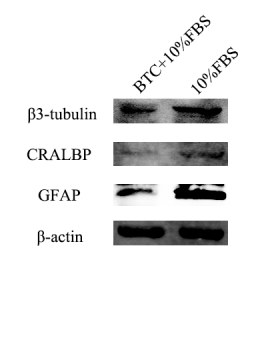


Figure. S10. Immunocytochemistry analysis of BTC pretreatment on RPC differentiation.


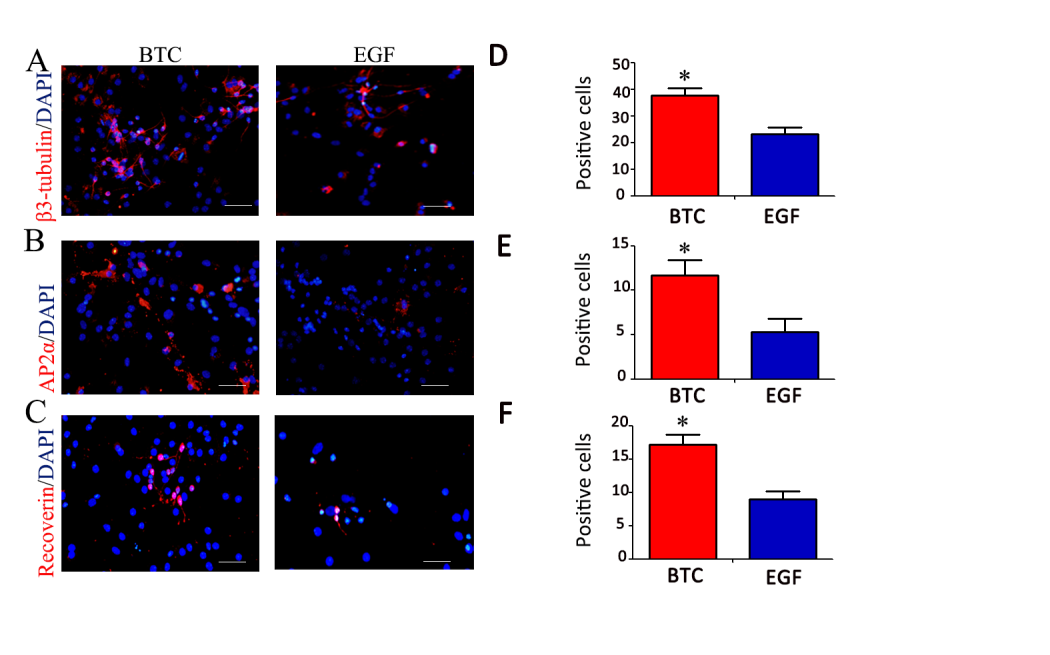


Figure. S11. Western blot analysis of BTC pretreatment combined with knockdown of BTC on RPC differentiation.


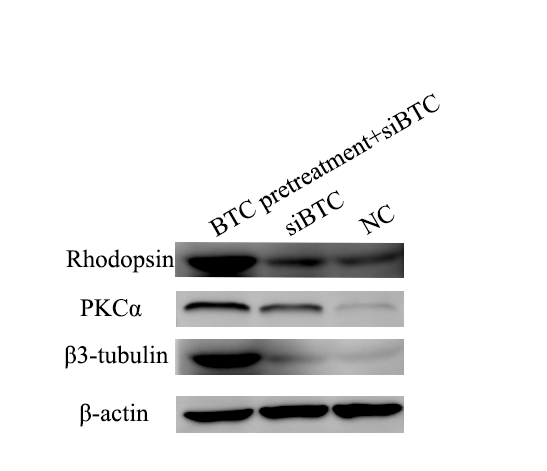


Supplementary Figure Legends

Figure. S1. CCK8 analysis of RPC proliferation in medium without any cytokines. RPC can proliferate in the first 24h while ceased to proliferate and even some died after that.

Figure. S2. Standard curve of CCK8 test on mRPCs.

Figure. S3. Phase picture of RPCs cultured in medium for proliferation and differentiation. A: RPCs were cultured in medium containing BTC or EGF, and phase picture were taken in day1,2 and 3, showing bigger cell clusters in BTC additive cultures than EGF additive cultures. Scale bars: 200 μm B: RPCs were cultured in medium containing BTC or EGF for 10 days and then BTC and EGF were withdrawed and medium containing 10%FBS were added. Phase pictures were taken at day10, 14 and 17, showing that cells ever treated with BTC have longer cell neurites. Scale bars: 100 μm

Figure. S4. qPCR analysis of P27 expression of RPCs cultured in medium containing BTC or EGF for 3 and 10 day respectively, showing an down-regulation of P27 expression in BTC group in comparison to EGF group.

Figure. S5. Antibody array detection of the receptor tyrosine kinase phosphorylation. RPCs grown in the presence of 20 ng/ml BTC or 20 ng/ml EGF for 20min were lysed, and the receptor tyrosine kinase phosphorylation was detected by an antibody array. The results showed that BTC caused stronger EGFR, ErbB2 and ErbB4 phosphorylation.

Figure. S6. LIVE/DEAD staining of RPC proliferate 3 days in standard medium with the presence of AG1478, AG825, LY294002 and PD98059 and the result showed no difference between AG1478, AG825, LY294002 and PD98059 groups and control group. Scale bars: 100 μm.

Figure. S7. Detection of the expression of Pax6 in BTC and EGF treated RPCs. (A): The expression levels of Pax6 (markers of RPCs) in the RPCs cultured with 20 ng/ml BTC or 20 ng/ml EGF were evaluated by qPCR analysis, and the results showed that there was no obvious difference between the two groups. (B): Western blot analysis of the expression levels of Pax6 in the RPCs cultured with 20 ng/ml BTC or 20 ng/ml EGF. (C): Immunocytochemistry analysis of the ratios of Pax6 -positive cells in the RPCs cultured with 20 ng/ml BTC or 20 ng/ml EGF. The results showed that there was no remarkable difference between the two groups. Scale bars: 25μm.

Figure. S8. Imunocytochemistry analysis of the differentiation state of the BTC or EGF treated cells in proliferation condition. Immunocytochemistry analysis showed that BTC or EGF treated RPCs didn’t express differentiation marker β3-tubulin, rhodopsin, CRX and CRALBP, implying that both BTC and EGF can keep RPCs in undifferentiated state.

Figure. S9. Western blot analysis were performed to clarify whether BTC additive in the differentiation medium directly (medium with 10% FBS but without EGF) will have an effect on RPC differentiation, showing that with BTC directly added in the differentiation medium, the differentiation ability of RPCs was weaken.

Figure. S10. Immunocytochemistry analysis of BTC pretreatment on RPC differentiation. Immunocytochemistry analysis showed that BTC pretreatment (10 days) can enhance RPC differentiation towards Rhodopsin, PKC-α and β3-tubulin positive cells. Scale bars: 50 μm.

Figure. S11. Protein expression levels were detected by combining pre-treatment with knockdown of BTC on day 10 (differentiation), showing that the expression of Rhodopsin, PKC-α and β3-tubulin in combining group were obviously up-regulated compared to siBTC group and NC group.
